# Association between dietary inflammatory index and epilepsy: findings from NHANES

**DOI:** 10.3389/fneur.2025.1599286

**Published:** 2025-05-30

**Authors:** Yike Zhu, Chuansen Lu

**Affiliations:** ^1^Department of Pulmonary and Critical Care Medicine, Hainan General Hospital, Hainan Affiliated Hospital of Hainan Medical University, Haikou, China; ^2^Department of Neurology, Hainan General Hospital, Hainan Affiliated Hospital of Hainan Medical University, Haikou, China

**Keywords:** NHANES, DII, epilepsy, cross-sectional study, diet

## Abstract

**Background:**

Inflammation plays a crucial role in the onset and progression of epilepsy. However, there is limited information regarding the relationship between diet-related inflammation and epilepsy. This study aimed to investigate the association between dietary inflammatory index (DII) and epilepsy.

**Methods:**

We conducted a cross-sectional analysis using data from the National Health and Nutrition Examination Survey (NHANES) 2013–2020. The DII scores were calculated and categorized into quartiles. Logistic regression was applied to assess the association between DII and epilepsy. Additionally, restricted cubic spline (RCS) analysis and subgroup analyses were performed.

**Results:**

The study included a total of 10,761 participants. After adjusting for age, gender, race, body mass index (BMI), smoking status, alcohol consumption, stroke, diabetes, and hypertension, a significant positive association was observed between DII and epilepsy in multivariable logistic regression (quartile 4 vs. 1, OR = 2.66, 95% CI 1.66–4.28, *p* < 0.001). The RCS analysis further confirmed a positive linear relationship between increasing DII scores and epilepsy risk (*p* for overall = 0.0007, *p* for nonlinear = 0.5128). Subgroup analyses showed a consistent association between DII and epilepsy across different subgroups.

**Conclusion:**

Elevated DII scores are associated with the risk of epilepsy. To improve epilepsy prevention and management, attention to dietary inflammation regulation is essential.

## 1 Introduction

Epilepsy is one of the most prevalent chronic neurological disorders and a significant cause of disability and mortality. With a global prevalence of 0.5–1% and a lifetime incidence of 1–3%, epilepsy affects nearly 70 million individuals worldwide (1–3). Existing therapies are predominantly based on pharmacological interventions; however, the majority of antiepileptic medications are insufficient in preventing seizures and protecting the brain (4), highlighting the pressing requirement for enhanced preventative and curative strategies.

More and more clinical and experimental evidence reveals that inflammation may play a critical role in the pathophysiology of seizure and epilepsy (5–7). Elevated levels of systemic inflammatory biomarkers, including interleukins (ILs), tumor necrosis factor (TNF), interferon (IFN), and procalcitonin (PCT), have been identified in patients with epilepsy (8–14). Systemic inflammation can lead to the disruption of the blood–brain barrier (BBB), thereby allowing peripheral toxic molecules and cytokine-producing immune cells to infiltrate, which promotes the occurrence of epilepsy (5, 15). Multiple researches have supported that antagonizing peripheral inflammation can reduce the severity of epilepsy, providing new strategies for the prevention and treatment of the condition (15–17).

Diet serves as an essential factor in modulating systemic inflammation within the body. Numerous studies have demonstrated that various nutrients, foods, and non-nutrient food components can regulate inflammation both acutely and chronically (18–20). Highly processed foods, refined grains, foods rich in saturated fatty acids and sodium, simple carbohydrates, and red processed meats are known to be pro-inflammatory. In contrast, vegetables, fruits, whole grains, legumes, low-fat dairy, fish, and foods rich in antioxidants (omega-3 fatty acids, flavonoids) exhibit anti-inflammatory properties (21–23). We propose that dietary interventions capable of modulating systemic inflammation may have a preventative effect on epilepsy.

Clearly, people’s diets are often diverse, rather than consisting of isolated intake of individual foods or food constituents. The inflammatory properties of individual foods are insufficient to assess the inflammation levels across various dietary patterns. Thus, it is essential to assess the dietary inflammatory potential in a comprehensive way. The Dietary Inflammation Index (DII) is a well-validated, reliable, and widely applied nutritional tool that assesses the inflammatory potential of an individual’s diet based on the effects of various dietary components on key inflammatory biomarkers, such as C-reactive protein (CRP), interleukin-6 (IL-6), and tumor necrosis factor-*α* (TNF-α). It has been shown to be associated with systemic inflammation (24). The DII was originally developed by Cavicchia et al. (25) and improved by Shivappa et al. (24). In recent decades, the DII has been evaluated in cancer, diabetes, cardiovascular disease, asthma, neurodevelopment, and mental health disorders (26). However, to our knowledge, the association between DII and epilepsy has not yet been researched. Therefore, in the present study, we utilized the cross-sectional data of the National Health and Nutrition Examination Surveys (NHANES) to explore the relationship between dietary inflammation and epilepsy, with the aim of providing more precise guidance for the prevention strategies of epilepsy.

## 2 Methods

### 2.1 Study population and ethics

NHANES, launched by National Center for Health Statistics (NCHS), is an ongoing, nationwide cross-sectional survey that collects health and nutrition information from the U. S. civilian noninstitutional population. All protocols received approval from the NCHS Ethics Review Board (ERB) and were performed in accordance with the Declaration of Helsinki, with all NHANES participants providing signed informed consent (publicly available on the web)^1^ (27). The cross-sectional data utilized in this study were sourced from the NHANES database, spanning the period from 2013 to March 2020. The data collection for this database was conducted by a team of trained professionals affiliated with the NHANES research initiative. After obtaining these data, we conducted the subsequent statistical analyses independently. Participants missing dietary and prescription medication data were excluded.

### 2.2 Calculation of the DII

The DII is calculated based on individual dietary components, requiring 45 dietary components in total (24). However, most studies analyze only a subset of these components. Shivappa et al. reported that the DII calculation retains its predictive ability even with fewer than 30 food parameters (28). The DII calculation formula is as follows:

For each dietary component, calculate the *Z*-score of individual intake:


$$Z\;\text{score}=\frac{\text{daily mean intake}-\text{global daily mean intake}}{\text{standard deviation}}$$

Convert the *Z*-score to a percentile score, which is then standardized to a range between −1 and 1:


Zscore′=(Zscore percentile score)×2−1

Multiply the standardized percentile score by the inflammatory effect score for each component, and then sum the scores for all components to obtain the individual’s overall DII score:


DII=∑(Zscore′×inflammation effect score)

A lower DII score indicates a more anti-inflammatory diet, while a higher DII score indicates a more pro-inflammatory diet (24).

Due to the limitations of NHANES data collection, this study, following the approach of other literature, used 28 dietary components for DII calculation (29–31). These components are protein, energy, carbohydrates, dietary fiber, total fat, saturated fat, monounsaturated fatty acids (MUFAs), polyunsaturated fatty acids (PUFAs), n-3 fatty acids, n-6 fatty acids, cholesterol, *β*-carotene, folate, vitamin A, vitamin B1, vitamin B2, niacin, vitamin B6, vitamin B12, vitamin C, vitamin D, vitamin E, magnesium, iron, zinc, selenium, caffeine, and alcohol. Dietary data were collected through 24-h dietary recall interviews by the NHANES Working Group on Nutrition Methods. Two separate dietary recalls were conducted in all participants: the first was a face-to-face interview at the Mobile Examination Center (MEC), and the second was completed via telephone 3 to 10 days later. This approach helps to provide a more comprehensive assessment of each participant’s dietary habits (32). To minimize the potential for recall bias, the dietary data from the two 24-h recalls were averaged.

### 2.3 Assessment of epilepsy

Epilepsy was defined by NHANES questionnaire data labeled “prescription medications.” In the study, participants who self-reported that their main reason for taking prescription medication in the past 30 days was “epilepsy and recurrent seizures” (International Classification of Disease, Tenth Revision, Clinical Modification [ICD-10-CM] Code: G40) were classified as having epilepsy (33, 34).

### 2.4 Covariates

Covariates were selected based on prior literature and biological plausibility. The demographic and questionnaire data were obtained through standardized questionnaires and face-to-face interviews, including gender (male, female), age (≤18, >18 years), race (Mexican American, non-Hispanic White, non-Hispanic Black, and other races), alcohol consumption, smoking status, and histories of stroke, diabetes, and hypertension. Based on total weekly alcohol consumption, Alcohol consumption was categorized based on total weekly intake as none, normal (1–14 drinks/week for males and 1–7 drinks/week for females), and heavy (≥15 drinks/week for males and ≥8 drinks/week for females). Smoking status was categorized as never, former, and current, with participants who had smoked at least 100 cigarettes in their lifetime defined as smokers. Physical examination was conducted by experienced medical staff in the MEC. Body mass index (BMI) data, calculated as weight (kg) divided by the square of height (m^2^), were used to estimate overweight/obesity status. Histories of stroke, diabetes, and hypertension can be defined based on self-reported previous diagnoses by a physician.

### 2.5 Handling of missing variables

To maximize the sample size and minimize bias from missing covariate data, we employed the multiple-imputation method for participants with incomplete covariate information. Missing values were imputed using chained equations with a 20-fold multiple imputation method. Supplementary Figure S1 presents the distribution of variables with missing data in our study.

### 2.6 Statistical analysis

We divided the study participants into four groups based on quartiles of the DII scores (Q1 to Q4) and compared differences in baseline characteristics across these quartiles. Categorical variables were presented as frequencies and percentages, whereas continuous variables were expressed as mean ± standard deviation (SD). Comparison of categorical and continuous variables were performed using the Pearson chi-squared test and Student’s *t*-test, respectively.

The association between DII and epilepsy was examined using logistic regression models, with odds ratios (OR) and 95% confidence intervals (95% CI) reported. In Model 1, no covariate was adjusted for; Model 2 was adjusted for age, gender, and race; Model 3 further included BMI, smoking status, alcohol consumption, stroke, diabetes, and hypertension. The first quartile (Q1) was designated as the reference group. Additionally, multivariate-adjusted (fully adjusted) restricted cubic spline (RCS) logistic regression analyses (choosing 4 knots, 5th, 35th, 65th, and 95th percentiles, respectively) were also conducted to examine the linear and dose–response associations between DII and epilepsy.

Furthermore, we selected covariates including age, gender, race, BMI, smoking status, alcohol consumption, stroke, diabetes, and hypertension for subgroup analyses to evaluate whether these covariates significantly interacted with the association between DII and epilepsy.

Finally, a sensitivity analysis was conducted, excluding participants with missing values for any variable, to verify the robustness of the results.

R software version 4.3.3^2^ was used for all statistical analyses. A *p*-value of less than 0.05 was considered statistically significant.

## 3 Results

### 3.1 Baseline characteristics of the study participants

**Figure 1:**
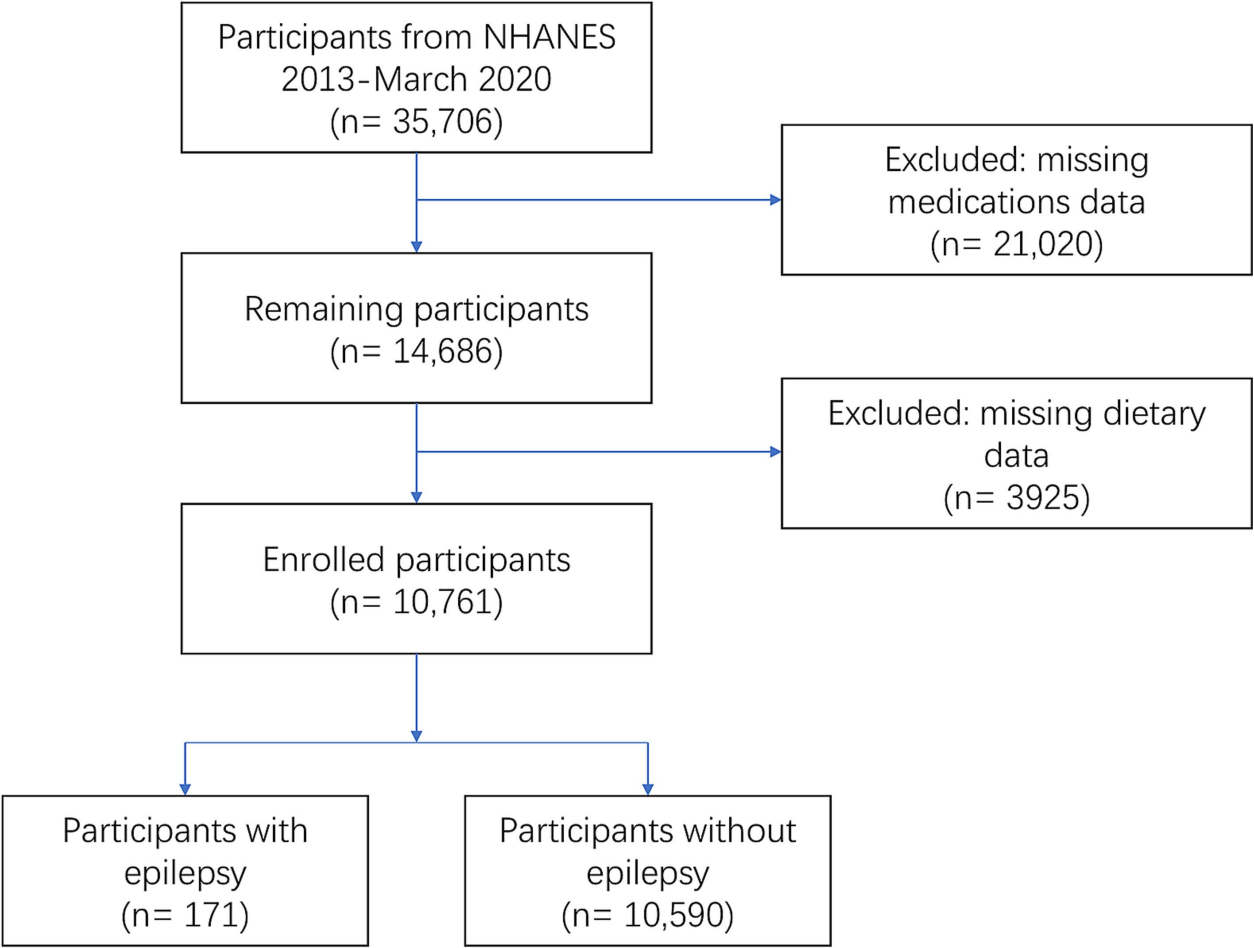
Flow diagram of study participants.

In this study, cross-sectional data of 35,706 participants from NHANES (2013-March 2020) were initially included. After manual data filtration, a total of 10,761 eligible participants were finally included in our analysis (Figure 1).

The baseline characteristics of all participants, classified into four groups by DII quartiles, are shown in Table 1. Compared with participants in the lowest quartile group (Q1), those with higher DII scores were more likely to be female, younger (≤18 years old), non-Hispanic black, current smokers, non-alcohol consumers, and have higher BMI, as well as a history of stroke and hypertension.

**Table 1:** Baseline characteristics of the study participants grouped by DII quartiles.

Characteristics	Quartiles of DII				
	Quartile 1	Quartile 2	Quartile 3	Quartile 4	*p*-value
*n*	2,691	2,689	2,691	2,690	
Gender, *n* (%)					<0.001
Female	1,182 (43.9)	1,337 (49.7)	1,563 (58.1)	1841 (68.4)	
Male	1,509 (56.1)	1,352 (50.3)	1,128 (41.9)	849 (31.6)	
Age, *n* (%)					<0.001
≤18 years	203 (7.5)	385 (14.3)	492 (18.3)	525 (19.5)	
>18 years	2,488 (92.5)	2,304 (85.7)	2,199 (81.7)	2,165 (80.5)	
Race, *n* (%)					<0.001
Mexican American	332 (12.3)	299 (11.1)	300 (11.1)	285 (10.6)	
Non−Hispanic Black	505 (18.8)	590 (21.9)	710 (26.4)	753 (28.0)	
Non−Hispanic White	1,227 (45.6)	1,203 (44.7)	1,147 (42.6)	1,120 (41.6)	
Others	627 (23.3)	597 (22.2)	534 (19.8)	532 (19.8)	
BMI, mean (SD)	29.09 (7.44)	29.05 (7.94)	29.25 (8.37)	29.67 (8.82)	0.022
Smoking status, *n* (%)					<0.001
Never	1,441 (57.6)	1,262 (54.0)	1,213 (54.5)	1,129 (51.3)	
Former	802 (32.1)	728 (31.1)	601 (27.0)	564 (25.6)	
Current	257 (10.3)	349 (14.9)	413 (18.5)	506 (23.0)	
Alcohol consumption, *n* (%)					<0.001
None	724 (31.0)	760 (34.5)	784 (37.1)	912 (45.1)	
Normal	1,372 (58.7)	1,258 (57.1)	1,141 (54.0)	1,001 (49.5)	
Heavy	241 (10.3)	186 (8.4)	188 (8.9)	109 (5.4)	
DII, mean (SD)	−0.94 (0.92)	0.91 (0.36)	2.06 (0.30)	3.31 (0.49)	<0.001
Stroke, *n* (%)					<0.001
Yes	113 (4.6)	139 (6.1)	133 (6.1)	206 (9.7)	
No	2,352 (95.4)	2,151 (93.9)	2044 (93.9)	1921 (90.3)	
Diabetes, *n* (%)					0.522
Yes	509 (18.9)	549 (20.4)	536 (19.9)	543 (20.2)	
No	2,182 (81.1)	2,138 (79.6)	2,152 (80.1)	2,147 (79.8)	
Hypertension, *n* (%)					<0.001
Yes	1,310 (51.8)	1,255 (52.6)	1,316 (57.3)	1,278 (56.2)	
No	1,221 (48.2)	1,131 (47.4)	981 (42.7)	994 (43.8)	

Results are shown as *n* (%) for binary variables, and as mean (standard deviation, SD) for continuous variables. BMI, body mass index.

Additionally, we also summarized the baseline characteristics of all individuals based on the presence of epilepsy (Supplementary Table S1).

### 3.2 Relationship between DII and epilepsy

**Figure 2:**
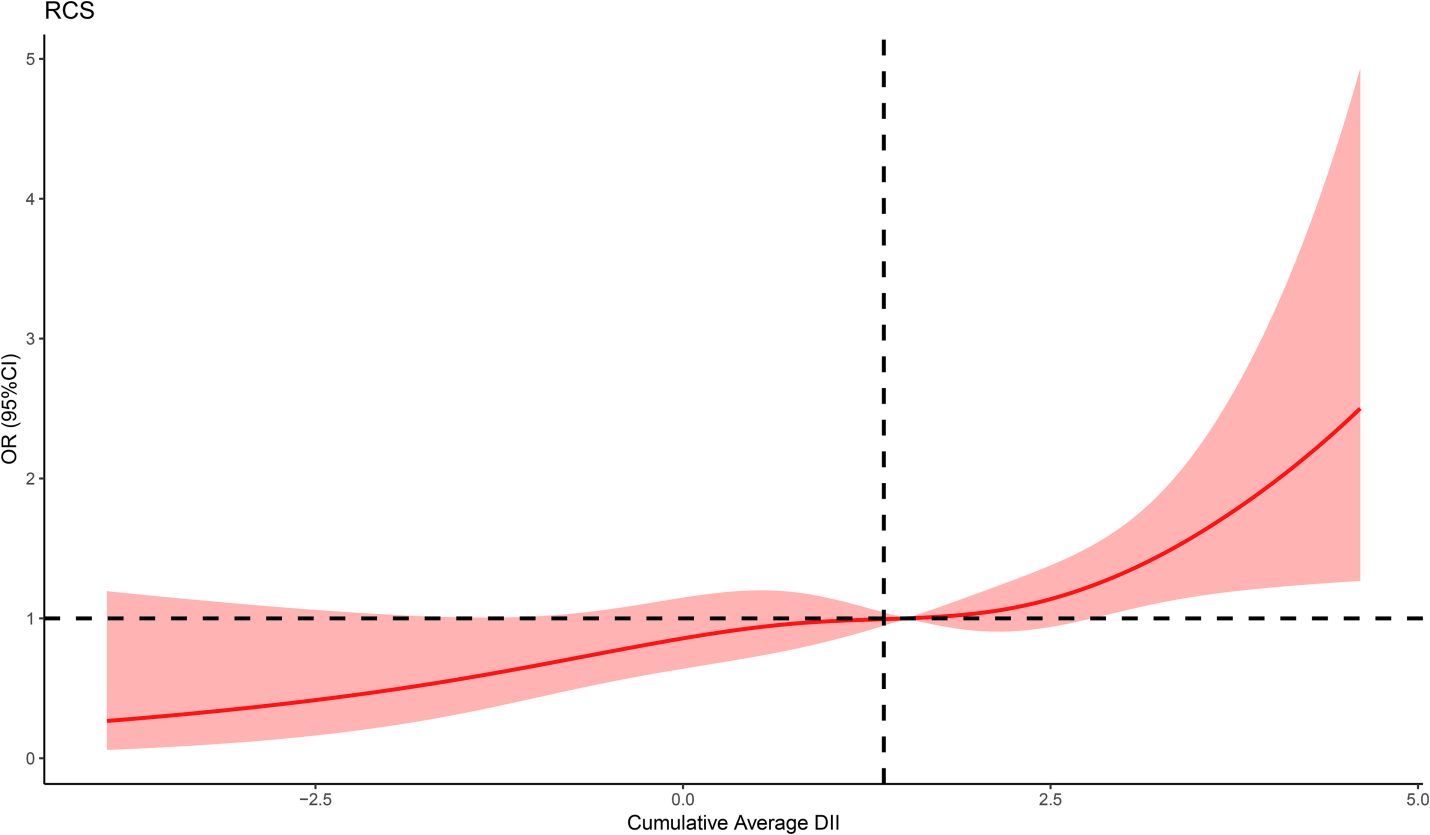
The RCS curve of the association between DII and epilepsy. RCS regression was adjusted for age, gender, race, BMI, smoking status, alcohol consumption, stroke, diabetes, and hypertension.

We constructed three logistic regression models to examine the association between DII and epilepsy, as presented in Table 2. In the non-adjusted model (Model 1), DII quartiles Q2 and Q4 were statistically significantly associated with epilepsy compared to Q1 (Q2: OR = 1.91, 95% CI 1.16–3.14, *p* = 0.011; Q4: OR = 2.42, 95% CI 1.47–3.96, *p* < 0.001). These associations remained significant in both Model 2 (Q2: OR = 1.87, 95% CI 1.14–3.07, *p* = 0.013; Q4: OR = 2.21, 95% CI 1.37–3.58, *p* = 0.001) and Model 3 (Q2: OR = 1.98, 95% CI 1.21–3.24, *p* = 0.007; Q4: OR = 2.66, 95% CI 1.66–4.28, *p* < 0.001). When DII was treated as a continuous variable, a 1 SD increase in DII was significantly associated with epilepsy across all models (Model 1: OR = 1.34, 95% CI 1.13–1.59, *p* < 0.001; Model 2: OR = 1.29, 95% CI 1.09–1.52, *p* = 0.003; Model 3: OR = 1.39, 95% CI 1.18–1.64, *p* < 0.001). In the fully adjusted RCS regression model, we observed a positive linear association between DII and epilepsy (*p* for overall = 0.0007, *p* for nonlinear = 0.5128) (Figure 2).

**Table 2:** Logistic regression analysis on the association between DII and epilepsy.

DII	Cases, *n* (%)	Model 1		Model 2		Model 3	
		OR (95% CI)	*p*	OR (95% CI)	*p*	OR (95% CI)	*p*
Quartiles							
Q1	24 (0.89%)	Reference	-	Reference	-	Reference	-
Q2	47 (1.75%)	1.91 (1.16, 3.14)	0.011	1.87 (1.14, 3.07)	0.013	1.98 (1.21, 3.24)	0.007
Q3	37 (1.38%)	1.50 (0.88, 2.54)	0.13	1.43 (0.85, 2.40)	0.02	1.55 (0.92, 2.60)	0.10
Q4	63 (2.34%)	2.42 (1.47, 3.96)	<0.001	2.21 (1.37, 3.58)	0.001	2.66 (1.66, 4.28)	<0.001
*p* for trend	-	0.002		0.006		<0.001	
Continuous							
Per 1 SD increase	-	1.34 (1.13, 1.59)	<0.001	1.29 (1.09, 1.52)	0.003	1.39 (1.18, 1.64)	<0.001

Model 1 was adjusted for none. Model 2 was adjusted for age, gender, and race. Model 3 was adjusted for age, gender, race, BMI, smoking status, alcohol consumption, stroke, diabetes, and hypertension. OR, odds ratio; CI, confidence interval; DII, dietary inflammation index; Q1, 1st quartile; Q2, 2nd quartile; Q3, 3rd quartile; Q4, 4th quartile; SD, standard deviation.

### 3.3 Subgroup analysis

**Figure 3:**
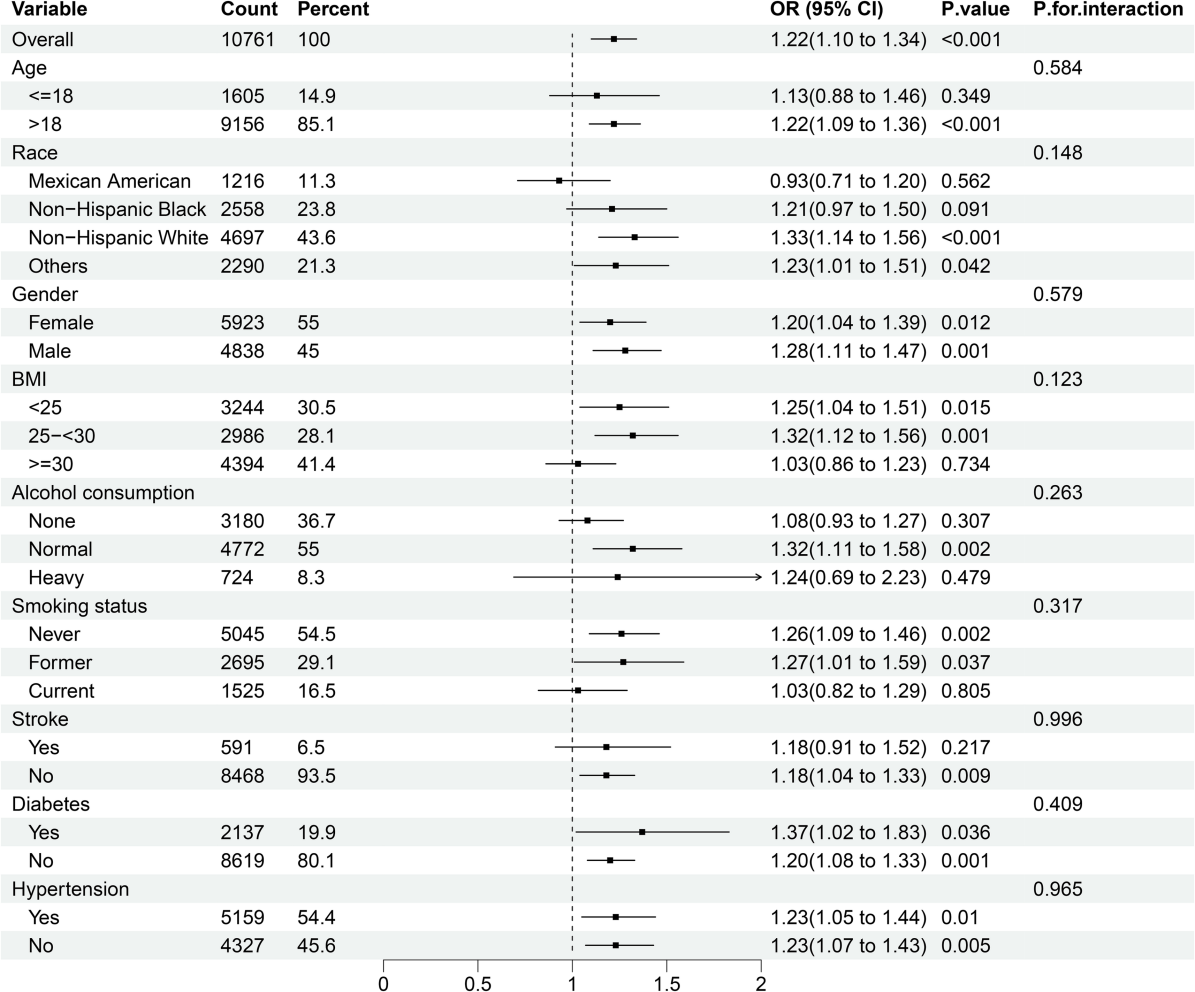
Subgroup analyses of the association between DII and epilepsy. Analyses were stratified for age (≤18 years, and >18 years), gender (female and male), race (Mexican American, non-Hispanic White, non-Hispanic Black, and other races), smoking status (never, former, and current), alcohol consumption (none, normal, and heavy), stroke (yes and no), diabetes (yes and no), and hypertension (yes and no).

We carried out subgroup analysis stratified by age (≤ 18 years, and > 18 years), gender (female and male), race (Mexican American, non-Hispanic White, non-Hispanic Black, and other races), BMI (<25, 25–<30, ≥30), smoking status (never, former, and current), alcohol consumption (none, normal, and heavy), stroke (yes and no), diabetes (yes and no), and hypertension (yes and no) to investigate whether the relationship between DII and epilepsy remained consistent across different subgroups (Figure 3). The results indicated a significant association between DII and epilepsy in most subgroups, while no significant interactions were observed that affected this relationship (all P for interaction > 0.05).

### 3.4 Sensitivity analysis

A sensitivity analysis was performed after excluding participants with missing values for any covariates (Supplementary Table S2). The results from both non-adjusted and adjusted models were consistent with the primary analysis, thereby confirming the stability and reliability of the findings.

## 4 Discussion

Research on the relationship between the DII and epilepsy remains limited. A study by Ding et al. demonstrated that adult epilepsy patients had higher DII scores compared to non-epileptic subjects (35). However, it did not include data from children under the age of 20, thereby limiting its generalizability. In contrast, our study collected a larger number of cases and included data from pediatric epilepsy patients. In our findings, logistic regression analysis revealed a positive association between high DII scores and epilepsy. Even after adjusting for other covariates, this relationship remained robust. Additionally, dose–response analysis showed a linear positive relationship. Stratified analysis indicated that DII was positively associated with epilepsy in most subgroups.

Although the research on the relationship between the DII and the occurrence and development of epilepsy is still scarce at present, the association between diet and epilepsy has long been a prominent research topic. A recent study by Zhang et al. investigated the association between the comprehensive dietary antioxidant index (CDAI) and epilepsy in the US population and found that a higher CDAI level corresponds to a lower risk of epilepsy, which suggests that a diet rich in antioxidants may help prevent epilepsy (34). He et al. reported that reduced antioxidant intake is associated with an increased risk of psychiatric comorbidities in epilepsy patients (36). Another study demonstrated that diet-derived circulating *β*-carotene significantly reduces epilepsy risk (37). Park et al. showed that naringin, a flavonoid found in grapefruit and citrus fruits, can reduce spontaneous recurrent seizures in a kainic acid-induced mouse model (38). Thus, these findings indicate that diet-related inflammation can influence epilepsy risk, providing a theoretical basis for our further research on the link between DII and epilepsy.

Several studies have demonstrated that pro-inflammatory diets can elevate systemic inflammation levels. D’Esposito et al. found that red meat consumption is associated with significant rises in IL-6, IL-8, and CRP (39). A study from the United States showed that after consuming an energy-dense, high-fat, fast-food–style meal, participants experienced a significant increase in IL-1β levels (40). An expanding body of evidence now indicates a close relationship between systemic inflammation and epilepsy onset. It has been reported that circulating inflammatory mediators, such as IL-6, TNF-*α*, and IL-1β, may impair tight junction regulation in brain endothelial cells, leading to heightened BBB permeability, enabling inflammatory mediators to infiltrate the central nervous system and trigger neuroinflammation (41). Additionally, another study revealed that blood monocytes can migrate to the brain through a compromised BBB and mediate neuroinflammation by differentiating into macrophages or microglia-like cells (42). Huang et al. also discovered that inducing systemic inflammation in mice led to TNFα-mediated brain vascular endothelial damage and astrocyte dysfunction, thereby raising the mice’s susceptibility to seizures (6). Therefore, the mechanism by which a high-DII diet increases epilepsy risk appears to be closely related to systemic inflammation.

Diet is well-recognized as a critical factor in shaping and influencing the structure and function of gut microbiota (43, 44). The Western diet, characterized by high levels of fat and cholesterol, is a primary driver of gut microbiota dysbiosis (45). Extensive evidence links changes in gut microbiota to epilepsy (46, 47). Medel-Matus et al. showed that disturbances in the intestinal microbiota of rats, particularly those associated with long-term stress, can increase vulnerability to epilepsy (48). Peng et al. identified a potential relationship between gut microbiota dysbiosis and the pathogenesis of drug-resistant epilepsy (49). Gómez-Eguílaz et al. demonstrated that probiotic therapy aimed at restoring gut microbiota balance can reduce seizure frequency and enhance quality of life in patients with drug-resistant epilepsy (50). Hence, we propose that DII influences epilepsy not only through systemic inflammation but also by modulating gut microbiota.

We further conducted the RCS regression analysis and found a significant positive linear association between DII and the incidence of epilepsy. In subgroup analyses stratified by age, race, gender, and other factors, no between-group differences in the association between DII and epilepsy were observed, which underscores the generalizability of our findings.

This study encompasses several advantages worth considering. Given the substantial sample size included, the study provides a dependable conclusion and ensures accurate statistical power. Additionally, our study utilized RCS analysis to further illustrate the positive linear association between the DII and epilepsy, which could provide novel insights for health policy decision-makers.

We acknowledge several limitations inherent in the present study. First, as a cross-sectional study, it cannot establish causality or temporal relationships between the DII and epilepsy. Second, recall bias may arise when obtaining dietary intake information through self-reporting. Third, the NHANES database does not explicitly differentiate between epilepsy subtypes or the severity of epilepsy. Detecting the connection between dietary-induced inflammatory status and various classifications of epilepsy remains a significant challenge. Fourth, although we have thoroughly screened numerous covariates to mitigate confounding bias, unidentified confounders may still exist and may not be explicitly recorded in the NHANES database.

## 5 Conclusion

In summary, our research indicates that the DII is closely associated with the risk of epilepsy. The association between the DII and epilepsy is linear and positive. Our findings offer preliminary evidence that may assist public health officials in developing practical strategies. However, more prospective studies are needed, and further investigation is required to explore the potential mechanism through which diet contributes to inflammation in epilepsy.

## Data Availability

Publicly available datasets were analyzed in this study. This data can be found here: https://www.cdc.gov/nchs/nhanes/.

## Footnotes

^1^ https://www.cdc.gov/nchs/nhanes/

^2^ http://www.R-project.org/
