# Supplementary material for: Association between dietary inflammatory index and epilepsy: findings from NHANES
Source: Front Neurol. 2025 May 30;16:1599286. doi: 10.3389/fneur.2025.1599286 (PMC12162894; doi:10.3389/fneur.2025.1599286)
Supplement: Supplementary file 1 [file Table_1.docx]

**Table S1.** Baseline characteristics of the study participants grouped by epilepsy status.

| **Characteristics** | **Non-Epilepsy** | **Epilepsy** | **P-value** |
| --- | --- | --- | --- |
| **n** | 10590 | 171 |  |
| **Gender, n (%)** | | | 0.305 |
| **Female** | 5836 (55.1) | 87 (50.9) |  |
| **Male** | 4754 (44.9) | 84 (49.1) |  |
| **Age, n (%)** | | | 0.031 |
| **≤18 years** | 1569 (14.8) | 36 (21.1) |  |
| **＞18 years** | 9021 (85.2) | 135 (78.9) |  |
| **Race, n (%)** | | | 0.919 |
| **Mexican American** | 1195 (11.3) | 21 (12.3) |  |
| **Non−Hispanic Black** | 2519 (23.8) | 39 (22.8) |  |
| **Non−Hispanic White** | 4625 (43.7) | 72 (42.1) |  |
| **Others** | 2251 (21.3) | 39 (22.8) |  |
| **BMI, mean (SD)** | 29.27 (8.17) | 29.14 (7.59) | 0.844 |
| **Smoking status, n (%)** | | | 0.112 |
| **Never** | 4971 (54.5) | 74 (53.6) |  |
| **Former** | 2662 (29.2) | 33 (23.9) |  |
| **Current** | 1494 (16.4) | 31 (22.5) |  |
| **Alcohol consumption, n (%)** | | | 0.002 |
| **None** | 3118 (36.4) | 62 (51.2) |  |
| **Normal** | 4718 (55.1) | 54 (44.6) |  |
| **Heavy** | 719 (8.4) | 5 (4.1) |  |
| **DII, mean (SD)** | 1.33 (1.66) | 1.83 (1.57) | <0.001 |
| **Stroke, n (%)** | | | <0.001 |
| **Yes** | 565 (6.3) | 26 (19.7) |  |
| **No** | 8362 (93.7) | 106 (80.3) |  |
| **Diabetes, n (%)** | | | 0.043 |
| **Yes** | 2114 (20.0) | 23 (13.5) |  |
| **No** | 8471 (80.0) | 148 (86.5) |  |
| **Hypertension, n (%)** | | | 0.221 |
| **Yes** | 5090 (54.5) | 69 (48.9) |  |
| **No** | 4255 (45.5) | 72 (51.1) |  |

Results are shown as n (%) for binary variables, and as mean (standard deviation, SD) for continuous variables. BMI, body mass index.
