# Supplementary material for: Association between dietary inflammatory index and epilepsy: findings from NHANES
Source: Front Neurol. 2025 May 30;16:1599286. doi: 10.3389/fneur.2025.1599286 (PMC12162894; doi:10.3389/fneur.2025.1599286)
Supplement: Supplementary file 2 [file Table_2.docx]

**Table S2.** Logistic regression analysis on the association between DII and epilepsy in sensitivity analysis

| **DII** | **Cases, n (%)** | **Model 1** | | **Model 2** | | **Model 3** | |
| --- | --- | --- | --- | --- | --- | --- | --- |
|  |  | **OR (95% CI)** | **P** | **OR (95% CI)** | **P** | **OR (95% CI)** | **P** |
| Quartiles | | | | | | | |
| Q1 | 18 (0.79%) | Reference | - | Reference | - | Reference | - |
| Q2 | 38 (1.78%) | 2.22 (1.28, 4.01) | 0.006 | 2.15 (1.24, 3.87) | 0.008 | 2.27 (1.31, 4.09) | 0.004 |
| Q3 | 21 (1.04%) | 1.33 (0.70, 2.55) | 0.4 | 1.21 (0.64, 2.32) | 0.6 | 1.32 (0.70, 2.51) | 0.4 |
| Q4 | 38 (1.96%) | 2.30 (1.29, 4.24) | 0.006 | 1.98 (1.12, 3.60) | 0.021 | 2.51 (1.45, 4.52) | 0.001 |
| P for trend | - | 0.042 | | 0.13 | | 0.014 | |
| Continuous | | | | | | | |
| Per 1 SD increase | - | 1.32 (1.07, 1.63) | 0.009 | 1.23 (1.01, 1.51) | 0.042 | 1.35 (1.11, 1.66) | 0.003 |

Model 1 was adjusted for none. Model 2 was adjusted for age, gender, and race. Model 3 was adjusted for age, gender, race, BMI, smoking status, alcohol consumption, stroke, diabetes, and hypertension. OR, odds ratio; CI, confidence interval; DII, dietary inflammation index; Q1, 1st quartile; Q2, 2nd quartile; Q3, 3rd quartile; Q4, 4th quartile; SD, standard deviation.
