# Supplementary figures and images for: Association between dietary inflammatory index and epilepsy: findings from NHANES
Source: Front Neurol. 2025 May 30;16:1599286. doi: 10.3389/fneur.2025.1599286 (PMC12162894; doi:10.3389/fneur.2025.1599286)

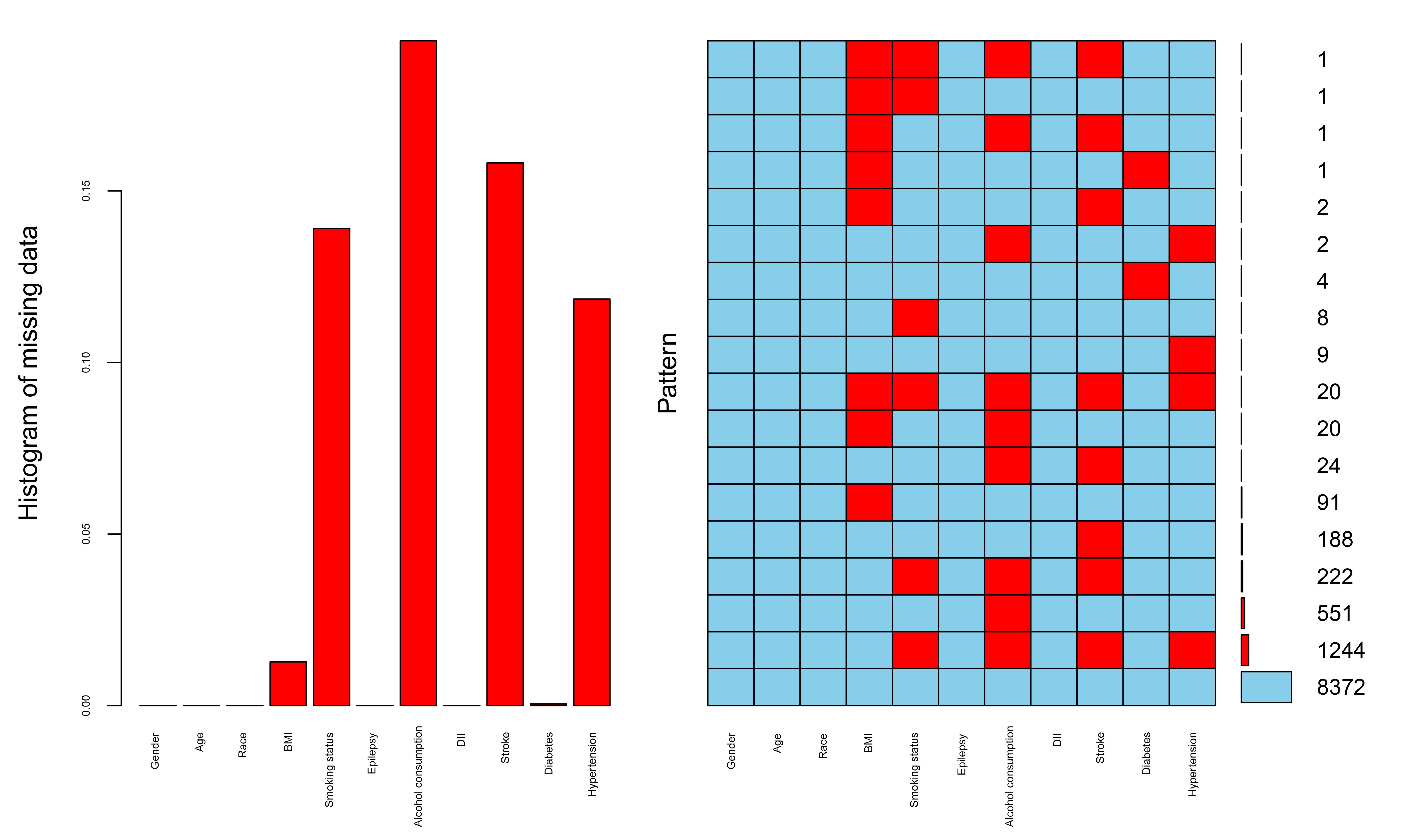

Supplement: Supplementary file 3 [file Image_1.tif]
